# A quasi‐Monte‐Carlo comparison of parametric and semiparametric regression methods for heavy‐tailed and non‐normal data: an application to healthcare costs

**DOI:** 10.1111/rssa.12141

**Published:** 2015-10-15

**Authors:** Andrew M. Jones, James Lomas, Peter T. Moore, Nigel Rice

**Affiliations:** ^1^ University of YorkUK; ^2^ ICON Health Economics and EpidemiologySydneyAustralia

**Keywords:** Healthcare costs, Health econometrics, Heavy tails, Quasi‐Monte‐Carlo methods

## Abstract

We conduct a quasi‐Monte‐Carlo comparison of the recent developments in parametric and semiparametric regression methods for healthcare costs, both against each other and against standard practice. The population of English National Health Service hospital in‐patient episodes for the financial year 2007–2008 (summed for each patient) is randomly divided into two equally sized subpopulations to form an estimation set and a validation set. Evaluating out‐of‐sample using the validation set, a conditional density approximation estimator shows considerable promise in forecasting conditional means, performing best for accuracy of forecasting and among the best four for bias and goodness of fit. The best performing model for bias is linear regression with square‐root‐transformed dependent variables, whereas a generalized linear model with square‐root link function and Poisson distribution performs best in terms of goodness of fit. Commonly used models utilizing a log‐link are shown to perform badly relative to other models considered in our comparison.

## 1 Introduction

The distribution of healthcare costs provides many challenges to the applied researcher: values are non‐negative (often with many observations with costs of 0), heteroscedastic, positively skewed and leptokurtic. Although these, or similar, challenges are found within many areas of empirical economics, the large interest in modelling healthcare costs has driven the development of an expanding array of estimation approaches and provides a natural context to compare methods for handling heavy‐tailed and non‐normal distributions. For an excellent review of statistical methods for the analysis of healthcare cost data with an emphasis on data collected alongside randomized trials, see Mihaylova *et al*. (2011). Econometric models of healthcare costs include applications to risk adjustment in insurance schemes (Van de Ven and Ellis, 2000), in devolving budgets to healthcare providers (e.g. Dixon *et al*. (2011)), in studies calculating attributable healthcare costs to specific health factors or conditions (Johnson *et al*., 2003; Cawley and Meyerhoefer, 2012) and in identifying treatment costs in health technology assessments (Hoch *et al*., 2002).

In attempting to capture the complex distribution of healthcare costs, two broad modelling approaches have been pursued. The first consists of flexible parametric models—distributions such as the three‐parameter generalized gamma and the four‐parameter generalized beta of the second kind distributions. This approach is attractive because of the range of distributions that these models encompass, whereas models with fewer parameters are inherently more restrictive, especially in regard to the assumptions that they impose on higher moments of the distribution (e.g. skewness and kurtosis). The second is the use of semiparametric models including extended estimating equations (EEEs), finite mixture models and conditional density approximation estimators. The EEE model adopts the generalized linear models (GLMs) framework and allows for the link and distribution functions to be estimated from data, rather than specified *a priori*. Finite mixture models introduce heterogeneity (both observed and unobserved) through mixtures of distributions. Conditional density approximation estimators are implemented by dividing the empirical distribution into discrete intervals and then decomposing the conditional density function into ‘discrete hazard rates’. Despite the burgeoning availability of healthcare costs data via administrative records, together with an increased necessity for policy makers to understand the determinants of healthcare costs and more, it is surprising that no previous study compares comprehensively the models belonging to these two strands of literature. In this paper we compare these approaches both with each other and against standard practice: linear regression on levels, and on square‐root and log‐transformations, of costs and GLMs.

Traditional Monte Carlo simulation approaches would not be appropriate for such an extensive comparison, as we are interested in a very large number of permutations of assumptions underlying the distribution of the outcome variable. In addition, such studies are prone to affording advantage to certain models arising from the chosen distributional assumptions that are used for generating data. Instead, using a large administrative database consisting of the population of English National Health Service (NHS) hospital in‐patient users for the year 2007–2008 (6164114 unique patients), we adopt a quasi‐Monte‐Carlo approach where regression models are estimated on observations from one subpopulation and evaluated on the remaining subpopulation. This enables us to evaluate the regression methods in a rigorous and consistent manner—while ensuring that results are not driven either by overfitting to rare but influential observations, or traditional Monte Carlo distributional assumptions—and are generalizable to hospital in‐patient services.

This paper compares and contrasts systematically these recent developments in semiparametric and fully parametric modelling both against each other and against standard practice. More strictly speaking, these recent developments are those that have featured in a Monte Carlo, cross‐validation or quasi‐Monte‐Carlo empirical comparative study. An example of a promising method that is not compared is the extension to GLMs that was proposed by Holly *et al*. (2011)—the fourth‐order pseudo‐maximum‐likelihood method—which has been applied to healthcare costs in Holly (2009). No comprehensive empirical comparison of these methods is currently present in existing literature and, given the number of choices that are available for modelling heavy‐tailed, non‐normal data, this study makes an important contribution towards forming a ranking of possible approaches (for a similar study comparing propensity score methods, see Huber *et al*. (2013)). The focus of this paper is the performance of these models in terms of predicting the conditional mean, given its importance in informing policy in healthcare and its prominence in comparisons between econometric methods in healthcare cost regressions. Under certain circumstances this is justified, for instance if the policy maker has a sufficiently large budget (Arrow and Lind, 1970). Other features of the distribution may be of interest (Vanness and Mullahy, 2007), especially when the policy maker has a smaller budget to allocate to healthcare. Given our focus, we analyse bias, accuracy and goodness of fit of forecasted conditional means. We find that no model performs best across all metrics of evaluation. Commonly used approaches—linear regression on levels of costs, linear regression on log‐transformed costs, the use of gamma GLMs with log‐link, and the use of the log‐normal distribution—are not among the four best performing approaches with any of our chosen metrics. Our results indicate that models that are estimated with a square‐root link perform much better than those with log‐ or linear link functions. We find that linear regression with a square‐root‐transformed dependent variable is the best performing model in terms of bias; the conditional density approximation estimator (using a multinomial logit) for accuracy and the Poisson GLM with square‐root link best in terms of goodness of fit.

## 2 Previous comparative studies

Various studies have compared the performance of regression‐based approaches to modelling healthcare cost data, where model performance is assessed on either actual costs (i.e. costs with an unknown true distribution) (Deb and Burgess, 2003; Veazie *et al*., 2003; Buntin and Zaslavsky, 2004; Basu *et al*., 2006; Hill and Miller, 2010; Jones *et al*., 2014) or simulated costs from an assumed distribution (Basu *et al*., 2004; Gilleskie and Mroz, 2004; Manning *et al*., 2005). Using actual costs preserves the true empirical distribution of cost data, and all of its complexities, whereas simulating costs provides a benchmark using the known parameters of the assumed distribution (classic Monte Carlo sampling) against which models can be compared.

Studies based on the classic Monte Carlo design are therefore ideally suited to assessing whether or not regression methods can fit data when specific assumptions, and permutations thereof, are imposed or relaxed. The complexities of the observed distribution of healthcare costs are such that a comprehensive comparison of modelling approaches would require an infeasibly large number of permutations of distributional assumptions used to generate data to make a classic Monte Carlo simulation worthwhile. Choosing a subset of the possible permutations of assumptions is prone to cause bias in the results in favour of certain methods. A reliance on actual data, as an alternative approach, requires large data sets so that forecasting is evaluated on sufficient observations to reflect credibly all of the idiosyncratic features of cost data. With this approach, however, it is difficult to assess exactly which aspect of the distribution of healthcare costs is problematic for each method under comparison.

### 2.1 Studies using cross‐validation approaches

With improvements in computational capacity, there have recently been several studies using large data sets to perform quasi‐Monte‐Carlo comparisons across regression models for healthcare costs. Quasi‐Monte‐Carlo comparisons divide the data into two groups, with samples repeatedly drawn from one group and models estimated, whereas the other group is used to evaluate out‐of‐sample performance (using the coefficients from the estimated models). In this section, we briefly review work that has implemented quasi‐Monte‐Carlo comparisons (Deb and Burgess, 2003; Jones *et al*.,^2014^, ^2015^) as well as discuss related approaches and important results.

Deb and Burgess (2003) examined several models to predict healthcare expenditures by using a quasi‐Monte‐Carlo approach with data from the US Department of Veterans Affairs comprised of approximately 3 million individual records. From within these observations a subgroup of 1.5 million individual records was used as an ‘estimation’ group and another subgroup of 1 million records formed a ‘prediction’ group. Their results highlight a trade‐off between bias and precision, and the need for caution surrounding the use of finite mixture models at smaller sample sizes. In terms of bias, they found that linear regression (on levels and square‐root‐transformed levels of costs) performs best, whereas in terms of accuracy models based on a gamma density have better performance. Jones *et al*. (2014) focused exclusively on parametric models and suggested the use of the generalized beta of the second kind model as an appropriate distribution for healthcare costs. Their quasi‐Monte‐Carlo design compared this distribution together with its nested and limiting cases, including the generalized gamma model. Using data from hospital episode statistics split into ‘estimation’ and ‘validation’ sets, they found little evidence that the performance of models varies with sample size, but they found variation between models in their ability to forecast mean costs, with the generalized gamma distribution the most accurate and the beta of the second kind distribution the least biased. Jones *et al*. (2015) also adopted the quasi‐Monte‐Carlo design but focused entirely on estimating and forecasting based on the cumulative distribution function and not on the conditional mean of the distribution.

Hill and Miller (2010) and Buntin and Zaslavsky (2004) also used cross‐validation techniques so that models are estimated on samples of data and evaluated on the remaining observations. Samples for estimation and the remaining data for evaluation differ across replications such that, unlike a quasi‐Monte‐Carlo design, individuals may fall into either the estimation sample or the validation sample at each replication. This approach is less data intensive and providing sufficient replications should produce sufficient information in the evaluation exercise to judge model performance. The approaches are similar in that they both replicate the sampling process to ensure that there is no ‘lucky split’ and guard against overfitting by evaluating out of sample. An alternative approach was considered in Veazie *et al*. (2003) where models were estimated on samples of observations belonging to 1992–1993 and evaluated on 1993–1994 observations. This is closer to the quasi‐Monte‐Carlo design and could potentially evaluate on data with a different underlying generating process, since they are from a different time period. Out‐of‐sample performance was also used as one metric in Basu *et al*. (2006) where they undertook tests of overfitting (Copas, 1983).

### 2.2 Recent developments in semiparametric and fully parametric modelling

Table 1 outlines the literature comparing regression models for healthcare costs as described above. As shown, there is no study that comprehensively and systematically evaluates all recent developments in approaches. In addition, any synthesis of the existing literature would be inconclusive in terms of which method is most appropriate for an application. Among the semiparametric methods, the EEE model has never been directly compared in a rigorous evaluation against any of the finite mixture models. They have both separately been compared against standard practice (transformed dependent variable regression and GLMs) in Basu *et al*. (2006), and in Hill and Miller (2010) and Deb and Burgess (2003) for EEE and finite mixture models respectively. The conditional density approximation estimator, so far, has not been compared with other healthcare cost regression models using actual data, although evidence from Monte Carlo studies suggests that it is a versatile approach (compared with standard practice methods) (Gilleskie and Mroz, 2004). Jones *et al*. (2014) introduced the use of the flexible parametric generalized beta of the second kind distribution with healthcare cost regressions and compared this against the generalized gamma distribution, which is a limiting case of the former. Given an increasing interest in modelling healthcare costs for resource allocation, risk adjustment and identifying attributable treatment costs, together with the burgeoning availability of data through administrative records, a comprehensive and systematic comparison of available approaches is timely. The results of this comparison will have resonance beyond healthcare costs and should be of interest to empirical applications to other right‐skewed, leptokurtic or heteroscedastic distributions such as income and wages.

**Table 1:** Models included in recent published comparative work

*Method*	*Studies using Monte Carlo*			*Studies using cross‐validation*				*Studies using quasi‐Monte‐*		
	*methods*							*Carlo methods*		
				*Veazie*	*Buntin and*	*Basu*	*Hill and*			
	*Basu*	*Gilleskie*	*Manning*	*et al*.	*Zaslaysky*	*et al*.	*Miller*	*Deb and*	*Jones*	*This*
	*et al*.	*and Mroz*	*et al*.	*(* 2003 *)*	*(* 2004 *)*	*(* 2004 *)*	*(* 2010 *)*	*Burgess*	*et al*.	*study*
	*(* 2004 *)*	*(* 2004 *)*	*(* 2005 *)*					*(* 2003 *)*	*(2013)*	
Linear regression				✓	✓		✓	✓		✓
Linear regression (logarithmic)			✓		✓	✓	✓	✓		✓
Linear regression (square root)				✓	✓			✓		✓
Log‐normal	✓				✓				✓	✓
Gaussian GLM					✓					†
Poisson					✓		✓			✓
Gamma	✓	✓	✓		✓	✓	✓	✓	✓	✓
EEE models						✓	✓			✓
Weibull	✓		✓						✓	‡
Generalized gamma			✓				✓		✓	✓
Generalized beta of the second kind									✓	✓
Finite mixture of gamma distributions								✓		✓
Conditional density estimator		✓								✓

†Not commonly used and problematic in estimation for our data in preliminary work. ‡A special case of generalized gamma and generalized beta of the second kind distributions which are included in our analysis.

## 3 Specification of models

We compare 16 models that are applicable to healthcare cost data. Each makes different assumptions about the distribution of the outcome (cost) variable. Each regression uses the same vector of covariates $X_{i}$, although the precise way in which they affect the distribution varies across models. The covariates included are age, age${}^{2}$, age${}^{3}$, gender, gender*age, gender*age${}^{2}$, gender*age${}^{3}$ and 24 morbidity markers indicating the presence or absence, coded 1 and 0 respectively, of one or more spells with any diagnosis within the relevant subset of version 10 international classification of diseases (ICD) codes (the 24 groupings were determined on the basis of clinical factors and initial letter of the ICD code; see the on‐line appendix A for more details). All models specify at least one linear index of covariates $X_{i}^{\prime }\beta$. In addition, linear regression methods with transformed outcome require assumptions surrounding the form of heteroscedasticity (modelled as a function of $X_{i}$), to retransform predictions onto the natural cost scale (Duan, 1983). Within the GLM family, we explicitly model the mean and variance functions as some transformation of the linear predictor (Blough *et al*., 1999). Fully parametric distributions, such as the gamma and beta family of models, require an assumption about the form of the entire distribution. In this paper, a single parameter is estimated as a function of the linear index. Finite mixture models allow for multiple densities, each a function of the covariates in linear form. For conditional density approximation estimator models, the empirical distribution of costs is divided into intervals, and functions of the independent variables predict the probability of lying within each interval.

Beginning with linear regression, we estimate three models by using ordinary least squares (OLS): the first is on the level of costs; the second and third use a log‐ and square‐root‐transformed dependent variable respectively (log‐transformation is more commonly used in the literature (Jones, 2011)). With these approaches, predictions are generated on a transformed scale, and it is necessary to calculate an adjustment to retransform predictions to their natural cost scale. This is done by applying a smearing factor, which varies according to covariates in the presence of heteroscedasticity (Duan, 1983). Residuals from the first regression of transformed healthcare cost against covariates are transformed by using the link function. Regressing the transformed residual against the covariates and taking these predicted values gives each observation's smearing factor.

Given the complications in retransformation in the presence of heteroscedasticity, researchers more frequently use methods that estimate on the natural cost scale and explicitly model the variance as a function of covariates. The dominant approach that achieves these aims is the use of GLMs (Blough *et al*., 1999). There are two components to a GLM: the first is a link function that relates the index of covariates to the conditional mean, and the second is a distribution function that describes the variance as a function of the conditional mean. These are estimated simultaneously, using pseudo‐ or quasi‐maximum‐likelihood, leading to estimates that are consistent provided that the mean function is correctly specified. Typically, the link function in applied work takes the form of a log‐ or square‐root function. In this paper we consider two types of distribution function, each a power function of the conditional mean. In the Poisson case, the variance is proportional to the conditional mean function of covariates and in the gamma case the variance is proportional to the conditional mean squared. Two of the combinations of link functions and distribution families are associated with commonly used distributions. In particular, the GLM with log‐link and gamma variance is commonly applied to healthcare costs, and the GLM with a log‐link and Poisson variance is associated with the Poisson model (see the discussion in Mullahy (1997)).

### 3.1 Flexible parametric models

Within the GLM and OLS approaches, much focus is placed on heteroscedasticity and the form that it takes. Recent developments in fully parametric modelling have been made where the modelling of higher moments, skewness and kurtosis is tackled explicitly. With this approach, the researcher estimates the entire distribution by using maximum likelihood, which requires that the distribution is correctly specified for consistent results. If the distribution is correctly specified, then estimates are efficient.

#### 3.1.1 Generalized gamma model

We estimate two models from within the gamma family, which have typically been used for durations, but also have precedent in the healthcare costs literature (Manning *et al*., 2005): the log‐normal and generalized gamma distributions. Each of these is estimated, using maximum likelihood, with a scale parameter specified as an exponential function of covariates, denoted $\text{exp}\;\left(X_{i}\prime \beta \right)$. The probability density function and conditional mean for the generalized gamma distribution are

(1) $$f\left(y_{i}|X_{i}\right)=\frac{\kappa {\left[\kappa^{-2}{\left\{y_{i}/\text{exp}\left(X_{i}^{\prime }\beta \right)\right\}}^{\kappa /\sigma }\right]}^{\kappa^{-2}}\text{exp}\left[-\kappa^{-2}{\left\{y_{i}/\text{exp}\left(X_{i}^{\prime }\beta \right)\right\}}^{\kappa /\sigma }\right]}{\sigma y_{i}\Gamma \left(\kappa^{-2}\right)},$$

(2) $$E\left(y_{i}|X_{i}\right)=\text{exp}\;\left(X_{i}^{\prime }\beta \right)\kappa^{2\sigma /\kappa }\frac{\Gamma (\kappa^{-2}+\sigma /\kappa )}{\Gamma (\kappa^{-2})}$$

where *σ* is a scale parameter, *κ* is a shape parameter and Γ(·) is the gamma function.

When *κ*→0 the generalized gamma distribution approaches the limiting case of the log‐normal distribution, for which the probability density function and conditional mean are

(3) $$f\left(y_{i}|X_{i}\right)=\frac{1}{\sigma y_{i}\surd \left(2\pi \right)}\text{exp}\;\;[\frac{-{\left\{\text{ln}\left(y_{i}\right)-X_{i}\prime \beta \right\}}^{2}}{2\sigma^{2}}],$$

(4) $$E\left(y_{i}|X_{i}\right)=\text{exp}\;\left(X_{i}^{\prime }\beta \right)\text{exp}\;\;(\frac{\sigma^{2}}{2}).$$

#### 3.1.2 Generalized beta of the second kind distribution

We also include the generalized beta of the second kind distribution, which has yet to be compared with a broad range of regression models (in Jones *et al*. (2014), beta‐type models were limited to comparison with gamma‐type distributions). Beta‐type models, like gamma‐type models, require assumptions about the form of the entire distribution. Until recently, they have been used largely in actuarial applications, as well as for the modelling of incomes (Cummins *et al*., 1990; Bordley *et al*., 1997). However, they have been suggested for use with healthcare costs because of their ability to model heavy tails, e.g. in Mullahy (2009), and they have been used with healthcare costs in Jones *et al*. (2014). We include the generalized beta of the second kind distribution, since all beta‐type (and gamma‐type) distributions are nested or limiting cases of this distribution. It therefore offers the greatest flexibility in terms of modelling healthcare costs among the duration models that are used here: see for example the implied restrictions on skewness and kurtosis (McDonald *et al*., 2013). The probability density function and conditional mean are

(5) $$f\left(y_{i}\right)=\frac{ay_{i}^{ap-1}}{b{\left(X_{i}\right)}^{ap}B\left(p,q\right){\left[1+{\left\{y_{i}/b(X_{i})\right\}}^{a}\right]}^{p+q}},$$

(6) $$E\left(y_{i}|X_{i}\right)=b\left(X_{i}\right)\left\{\frac{\Gamma (p+1/a)\Gamma (q-1/a)}{\Gamma (p)\Gamma (q)}\right\}$$

where *a* is a scale parameter, *p* and *q* are shape parameters and *B*(*p*,*q*)=Γ(*p*) Γ(*q*)/Γ(*p*+*q*) is the beta function.

We parameterize the generalized beta of the second kind distribution with the scale parameter *b* as two different functions of covariates: a log‐link and a square‐root link.

### 3.2 Semiparametric methods

#### 3.2.1 Extended estimating equations

A flexible extension of GLMs has been proposed by Basu and Rathouz (2005) and Basu *et al*. (2006), which is known as the EEE model. It approximates the most appropriate link by using a Box–Cox function, where *λ*=0 implies a log‐link and *λ*=0.5 implies a square‐root link:

(7) $$E\left(y_{i}|X_{i}\right)={\left(\lambda X_{i}^{\prime }\beta +1\right)}^{1/\lambda }$$

as well as a general power function to define the variance with constant of proportionality $\theta_{1}$ and power $\theta_{2}$:

(8) $$\text{var}\left(y_{i}|X_{i}\right)=\theta_{1}E{\left(y_{i}|X_{i}\right)}^{\theta_{2}}.$$

Suppose that the distribution of the outcome variable is unknown but has mean and variance nested within equations (7) and (8). An incorrectly specified GLM mean function, where common usage of GLM mean functions is limited to standard forms such as log‐ and square‐root link functions, yields biased and inconsistent estimates, whereas estimates from EEE models should be unbiased, provided that the specification of regressors is correct. A well‐specified mean function combined with an incorrectly specified distribution form will be inefficient compared with EEE models. If the distribution is known to be a specific GLM form, the EEE model is less efficient than the appropriate GLM, but both are unbiased.

#### 3.2.2 Finite mixture models

Finite mixture models have been employed in health economics to allow for heterogeneity both in response to observed covariates and in terms of unobserved latent classes (Deb and Trivedi, 1997). Heterogeneity is modelled through a number of components, denoted *C*, each of which can take a different specification of covariates (and shape parameters, where specified), written as $f_{j}\left(y_{i}X_{i}\right)$, and where there is a parameter for the probability of belonging to each component, $\pi_{j}$. The general form of the probability density function of finite mixture models is

(9) $$f\left(y_{i}|X_{i}\right)=\sum_{j}^{C}\pi_{j}f_{j}\left(y_{i}|X_{i}\right).$$

We use two gamma distribution components in our comparison. Preliminary work showed that models with a greater number of components led to problems with convergence in estimation. Empirical studies such as Deb and Trivedi (1997) provide support for the two‐components specification for healthcare use. In one of the models used, we allow for log‐links in both components (10), and in the other we allow for a square‐root link (11). In both, the probability of class membership is treated as constant for all individuals and a shape parameter $\alpha_{j}$ is estimated for each component:

(10) $$f_{j}\left(y_{i}|X_{i}\right)=\frac{y_{i}^{\alpha_{j}}}{y_{i}\Gamma \left(\alpha_{j}\right)\text{exp}{\;(X_{i}^{\prime }\beta_{j})}^{\alpha_{j}}}\text{exp}\;\{-\frac{y_{i}}{\text{exp}\;(X_{i}^{\prime }\beta_{j})}\},$$

(11) $$f_{j}\left(y_{i}|X_{i}\right)=\frac{y_{i}^{\alpha_{j}}}{y_{i}\Gamma \left(\alpha_{j}\right){\left(X_{i}^{\prime }\beta_{j}\right)}^{2\alpha_{j}}}\text{exp}\;\{-\frac{y_{i}}{{\left(X_{i}^{\prime }\beta_{j}\right)}^{2}}\}.$$

The conditional mean is given for the log‐link specification and for the square‐root link by equations (12) and (13) respectively:

(12) $$E\left(y_{i}|X_{i}\right)=\sum_{j}^{C}\pi_{j}\alpha_{j}\text{exp}\left(X_{i}^{\prime }\beta_{j}\right),$$

(13) $$E\left(y_{i}|X_{i}\right)=\sum_{j}^{C}\pi_{j}\alpha_{j}{\left(X_{i}^{\prime }\beta_{j}\right)}^{2}.$$

Unlike the models in the previous section, this approach can allow for a multimodal distribution of costs. In this way, finite mixture models represent a flexible extension of parametric models (Deb and Burgess, 2003). Using increasing numbers of components, it is theoretically possible to fit any distribution, although in practice researchers tend to use few components (two or three) and achieve good approximation to the distribution of interest (Heckman, 2001).

#### 3.2.3 Conditional density approximation estimators

Finally, we use two additional models that are applications of the conditional density approximation estimator that was outlined in Gilleskie and Mroz (2004). Their method is an extension of the two‐part model that is frequently used to deal with zero costs, in that the range of outcome variable is divided into *Q* parts (or intervals), where the mean (of observations to be used in estimation) within interval *j* (*j*=1,…,*Q*) is ${\bar{y}}_{j}$ and the lower and upper threshold values are $y_{j-1}$ and $y_{j}$ respectively (where $y_{0}$ is equal to the lowest observed cost and $y_{Q}$ is equal to the highest observed cost). The probability that an observation falls into interval *j* can be written as

(14) $$p_{ij}\left(X_{i}\right)=P\left(y_{j-1}\leq y_{i}<y_{j}|X_{i}\right)=\int_{y_{j-1}}^{y_{j}}f\left(y_{i}|X_{i}\right)dy_{i}.$$

The density function is then approximated by *Q* ‘discrete hazard rates’, defined as the probability of lying in interval *j* conditionally on not lying in intervals 1,…,*j*−1 and written as $\lambda (j,X_{i})$:

(15) $$\lambda \left(j,X_{i}\right)=P\left(y_{j-1}⩽y_{i}<y_{j}|X_{i},y_{i}⩾y_{j-1}\right)=\frac{\int_{y_{j-1}}^{y_{j}}f\left(y_{i}|X_{i}\right)dy_{i}}{1-\int_{y_{0}}^{y_{j-1}}f\left(y_{i}|X_{i}\right)dy_{i}}.$$

The effect of covariates can vary smoothly, or discontinuously, across intervals depending on how the model is specified: with the most flexible case using a separate model for each interval's hazard rate. We assume that only the probability of lying within an interval depends on covariates, and that the mean value of the outcome variable, for a given interval, does not vary with covariates. The conditional mean function is therefore obtained by using

(16) $$E\left(y_{i}|X_{i}\right)=\sum_{j=1}^{Q}p_{ij}\left(X_{i}\right){\bar{y}}_{j}.$$

One of the main benefits of this approach is the flexibility that is afforded with respect to the intervals that are used. There is flexibility in terms of the number of intervals, and where the boundaries between them are placed, as well as the degree to which the ‘discrete hazard rates’ are estimated separately for each interval. Within our illustration, we use 15 equally sized intervals across all samples. Gilleskie and Mroz (2004) in their application to healthcare costs found that between 10 and 20 intervals result in a good approximation, based on an adjusted log‐likelihood to guard against overfitting, and we found that 15 intervals resulted in good convergence performance in preliminary work. In practice, a researcher would experiment with different intervals and compare model performance to decide on the specification. Having decided on the intervals to be used, we use a multinomial logit specification and an ordered logit specification to model the probabilities of lying within each interval. This differs from the less parametric single‐logit specification that was adopted in Gilleskie and Mroz (2004), which is more computationally demanding, and instead uses an approach similar to that of Han and Hausman (1990). The multinomial logit specification is similar to running a separate logit model for each ‘discrete hazard rate’, whereas the ordered logit specification is analogous to allowing the discrete hazard rate to vary discontinuously for each interval but with no discontinuity in the effects of covariates. Fully adhering to Gilleskie and Mroz (2004) would allow the data to determine how flexibly to estimate the discrete hazard rates; once again our implementation is a simpler approach which approximates their method:

(17) $$p_{ij}\left(X_{i}\right)=\frac{\text{exp}\;(X_{i}^{\prime }\beta_{j})}{\sum_{l=1}^{Q}\text{exp}\;(X_{i}^{\prime }\beta_{l})}$$

where $\beta_{1}=0$ to normalize for estimation purposes

(18) $$p_{ij}\left(X_{i}\right)=\frac{\text{exp}\;(\psi_{j}-X_{i}^{\prime }\beta )}{1+\text{exp}\;(\psi_{j}-X_{i}^{\prime }\beta )}-p_{ij-1}$$

where $\psi_{j}$ represents the estimated threshold value for each category from the ordered logit model, $p_{i0}=0$ and $p_{i15}=1-p_{i14}$, so we estimate only 14 threshold values (in our application *Q*=15).

Conditional means from these models are calculated as in equation (16), where the probabilities $p_{ij}$ are calculated by using equation (17) for the multinomial logit specification and equation (18) for the ordered logit specification.

## 4 Data and choice of variables

Our study uses individual level data from the English hospital episode statistics (for the financial year 2007–2008). This data set contains information on all in‐patient episodes, out‐patient visits and accident and emergency department attendances for all patients admitted to English NHS hospitals (Dixon *et al*., 2011). For our study, we exclude spells which were primarily mental or maternity healthcare, as well as private sector spells. This data set was compiled as part of a wider project considering the allocation of NHS resources to primary care providers. Since much mental healthcare is undertaken in the community and with specialist providers, and hence is not recorded in the hospital episode statistics, the data are incomplete, and also since healthcare budgets for this type of care are constructed by using separate formulae. Maternity services are excluded since they are unlikely to be heavily determined by morbidity characteristics, and accordingly for the setting of healthcare budgets are determined by using alternative mechanisms. The hospital episode statistics database is a large administrative data set collected by the Health and Social Care Information Centre (now named the NHS Information Centre), with our data set comprising 6164114 separate observations, representing the population of hospital in‐patient healthcare users for the year 2007–2008. Since data are taken from administrative records, we have information only on users of in‐patient NHS services, and therefore we can only model strictly positive costs (0s are typically handled by a two‐part specification and the main challenge is to capture the long and heavy tail of the distribution rather than the 0s).

The cost variable that is used throughout is individual patient annual NHS hospital cost for all spells finishing in the financial year 2007–2008. To cost utilization of in‐patient NHS facilities, tariffs from 2008–2009 (reference costs for 2005–2006, which were the basis for the tariffs from 2008–2009, were used when 2008–2009 tariffs were unavailable) were applied to the most expensive episode within the spell of an in‐patient stay (following standard practice for costing NHS activity). Then, for each patient, all spells within the financial year were summed. The data are summarized in Table 2.

**Table 2:** Descriptive statistics for hospital costs

*Statistic*	*Results for*	*Results for*	*Results for*
	*full data set*	*estimation set*	*validation set*
*N*	6164114	3082057	3082057
Mean	£2610	£2610	£2610
Median	£1126	£1126	£1126
Standard deviation	£5088	£5090	£5085
Skewness	13.03	12.94	13.13
Kurtosis	36318	347.06	379.36
Maximum	£604701	£476458.3	£604701
99th percentile	£19015	£19074	£18955
95th percentile	£8956	£8943	£8969
90th percentile	£6017	£6010	£6025
75th percentile	£2722	£2721	£2722
25th percentile	£610	£610	£610
10th percentile	£446	£446	£446
5th percentile	£407	£407	£407
1st percentile	£347	£347	£347
Minimum	£217	£217	£217

The challenges of modelling cost data are clearly observed in Table 2: the observed costs are heavily right hand skewed, with the mean far in excess of the median, and are highly leptokurtic. Placement in the estimation and validation subsets was determined by a random split and as seen in Table 2 there are only small differences in the summary statistics of the sets of observations. In particular, the ‘lumpy’ nature of the data—due to many mass points arising from the data‐generating process—can be seen from the number of percentiles that are the same across both subsets.

**Figure 1:**
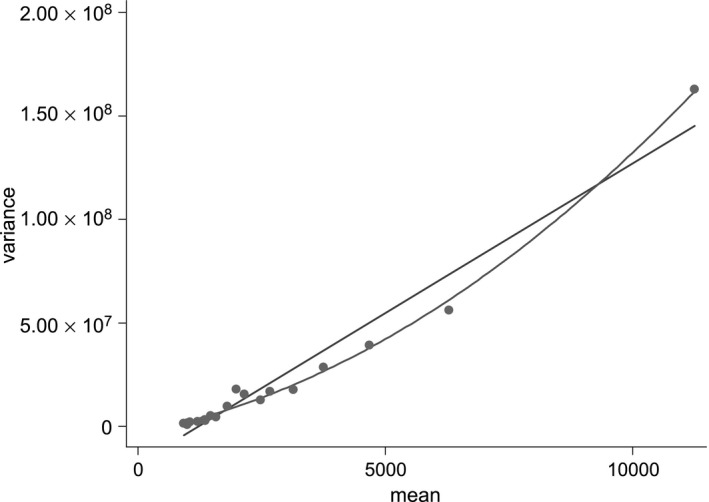
Variance against mean for each of the 20 quantiles of the linear index of covariates: the data were divided into 20 subsets by using the deciles of a simple linear predictor for healthcare costs with the set of regressors introduced later; the figure plots the means and variances of actual healthcare costs for each of these subsets, with fitted linear and quadratic trends

We construct a linear index of covariates (by regressing the outcome variable on the set of covariates that we include in our regression models by using OLS) and divide the data into quantiles according to this, to analyse conditional (on *X*) distributions of the outcome variable. First, we plot the variances of each quantile against their means (Fig. 1). This gives us a sense both of the nature of heteroscedasticity and of feasible assumptions relating these aspects of the distribution. From Fig. 1, we can see that there is evidence against homoscedasticity (where there would be no visible trend), and evidence for some relationship between the variance and the mean. A similar analysis can be carried out for higher moments of the distribution, plotting the kurtosis of each quantile against their skewness. Parametric distributions impose restrictions on possible skewness and kurtosis: one‐parameter distributions are restricted to a single point (for example a normal distribution imposes a skewness of 0 and a kurtosis of 3), two‐parameter distributions allow for a locus of points to be estimated and distributions with three or more parameters allow for spaces of possible skewness and kurtosis combinations. For further details see Holly and Pentsak (2006), Pentsak (2007), McDonald *et al*. (2013) and Jones *et al*. (2015).

All the models in the quasi‐Monte‐Carlo comparison use a specified vector of covariates and have at least one linear index of these. This vector mirrors the practice in the literature regarding comparing econometric methods for healthcare costs, allowing models to control for age (as well as age squared and age cubed), gender (interacted fully with age terms) and morbidity characteristics (from ICD classifications). Morbidity information is available through the hospital episode statistics data set, adapted from the ICD version 10 chapters (World Health Organization, 2007)—see the on‐line appendix A for further details. Each of the 24 morbidity markers indicates the presence or absence, coded 1 and 0 respectively, of one or more spells with any diagnosis within the relevant subset of ICD version 10 chapters, during the financial year 2007–2008 (see appendix A). We do not use a fully interacted specification, since morbidity is modelled with a separate intercept for the presence of each type of diagnosis (and not interacted with age or gender). However, we do allow for interactions between age and its higher orders and gender. This means that we are left with a specification that is close to those used in the comparative literature as well as a parsimonious version of the set of covariates that are used to model costs in person‐based resource allocation in England, as in, for example, Dixon *et al*. (2011). In addition, making the specification less complicated aids computation and results in fewer models failing to converge.

## 5 Methodology

### 5.1 Quasi‐Monte‐Carlo design

**Figure 2:**
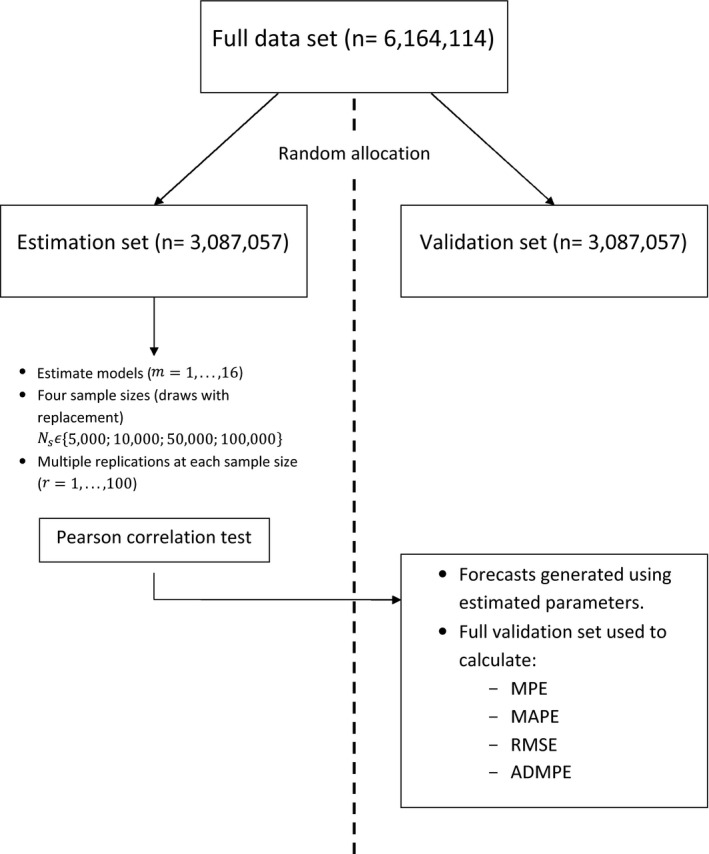
Diagram setting out the study design

By using the hospital episode statistics data, we have access to a large amount of observations representing the whole population of English NHS in‐patient costs. To exploit this, we use a quasi‐Monte‐Carlo design similar to that of Deb and Burgess (2003). The population of observations (6164114) is randomly divided into two equally sized subpopulations: an estimation set (3082057) and a validation set (3082057). From within the estimation set we randomly draw, 100 times with replacement, samples of size $N_{s}$ ($N_{s}\in 5000,10000,50000,100000$). The models are estimated on the samples and performance then evaluated on the sample drawn from both the estimation set and the full validation set. Using a split sample to evaluate models has precedent in the comparative literature on healthcare costs; see Duan *et al*. (1983) and Manning *et al*. (1987). Fig. 2 illustrates our study design in the form of a diagram: note that the subscript *m* denotes the model used, $N_{s}$ the sample size used and *r* the replication number.

To execute this quasi‐experimental design, we automate the model selection process for each approach: for instance, with the conditional density approximation estimator, we specify a number of bins to be estimated, *a priori*, rather than undergoing the investigative process that was outlined in Gilleskie and Mroz (2004). Similarly, all models have been automated to some extent, since we set *a priori* the specification of regressors (all models), the parameters that vary with covariates (generalized gamma and generalized beta of the second kind models) and the number of mixtures to model (finite mixture models). Our specification of regressors was based on preliminary work, which showed that alternative specifications, including the use of a count of the number of morbidities, give similar results, but with worse convergence performance.

### 5.2 Evaluation of model performance

#### 5.2.1 Estimation sample

Researchers modelling healthcare costs will typically carry out multiple tests to establish the reliability of their model specification. These tests are carried out in sample and help to inform the selection of models that will then be used for predictive purposes. They are commonly used to build the specification of the ‘right‐hand side’ of the regression: the covariates used and interactions between them. In addition, researchers working with healthcare costs use these tests to establish the appropriate link function between covariates and expected conditional mean, and other assumptions about functional form. We include results from the Pearson correlation coefficient test, which is simple to carry out and has intuitive appeal. Results from Pregibon link, Ramsey RESET and modified Hosmer–Lemeshow tests, in preliminary work, were found to display the same pattern as the Pearson correlation coefficient test, but with smaller variation in rejection rates across the different models. To carry out the Pearson correlation coefficient test, residuals (computed on the raw cost scale) are regressed against predicted values of cost. If the slope coefficient on the predicted costs is significant, then this implies a detectable linear relationship between the residuals and the covariates, and so evidence of model misspecification.

#### 5.2.2 Validation set

We use our models to estimate forecasted mean healthcare costs over the year for individuals (${\hat{y}}_{i}^{V}=\hat{E(y_{i}^{V}|X_{i}^{V})}$— computed by using coefficients from models estimated on the estimation set (for example for linear regression $E\left(\hat{y_{i}^{V}|X_{i}^{V}}\right)={\hat{\alpha }}^{E}+\hat{\beta }{}^{E}X_{i}^{V}$)—where V denotes that the observation is from the validation set, and we evaluate performance on metrics designed to reflect the bias (mean prediction error (MPE), ${\text{MPE}}_{msr}=\Sigma \left(y_{i}-{\hat{y}}_{i}\right)/N_{s}$), accuracy (mean absolute prediction error (MAPE), ${\text{MAPE}}_{msr}=\Sigma |y_{i}-{\hat{y}}_{i}|/N_{s}$) and goodness of fit (root‐mean‐square error (RMSE), ${\text{RMSE}}_{msr}=\surd \left\{\Sigma {\left(y_{i}-{\hat{y}}_{i}\right)}^{2}/N_{s}\right\}$) of these forecasts. The MPE can be thought of as measuring the bias of predictions at an aggregate level, where positive and negative errors can cancel each other out, whereas the MAPE is a measure of the accuracy of individual predictions. The RMSE is similar to the MAPE in that positive and negative errors do not cancel out; however, larger errors count for disproportionately more, since they are squared. In addition, we evaluate the variability of bias across replications (absolute deviations of the mean prediction error (ADMPE), ${\text{ADMPE}}_{msr}=\left|{\text{MPE}}_{msr}-\Sigma_{r=1}^{R}{\text{MPE}}_{msr}/R\right|$). These are all evaluated on the full validation set, where *m* denotes the model used, *s* the sample size used and *r* the replication.

Only replications where all 16 models are successfully estimated on the sample are included for evaluation, and model performance according to each criterion is calculated as an average over all included replications, e.g. ${\text{MPE}}_{ms}=\Sigma_{r=1}^{R}{\text{MPE}}_{msr}/R$. All models estimated successfully every time, except for the CDEM and EEE models. CDEM could not be estimated on two of the 100 replicates with samples of 5000 observations. The EEE models could not be estimated on four, four, six and four of the 100 replicates with sample sizes of 5000, 10000, 50000 and 100000 observations respectively.

To obtain a greater insight into the performance of different distributions, we evaluate forecasted conditional means at different values of the covariates. In practice this is done by partitioning the fitted values of costs into deciles. We assess the MPE and MAPE for deciles of predicted costs, since there is concern that models perform with varying success at different points in the distribution. Models designed for heavy tails, for instance, might be expected to perform better in predicting the biggest costs. This also represents a desire to fit the distribution of costs for different groups of observations according to their observed covariates.

We combine the results that we obtain from different sample sizes $N_{s}$ and attempt to find a pattern in the way in which models perform as the sample size varies. To do this we construct response surfaces (as in, for example, Deb and Burgess (2003)). These are polynomial approximations to the relationship between the statistics of interest and the sample size of the experiment, $N_{s}$. For our purposes, we estimate the following regression for each model and for each metric of performance (illustrated below for the MPE):

(19) $${\text{MPE}}_{msr}=\alpha_{m}^{\mathrm{MPE}}+\beta_{m}^{\mathrm{MPE}}\frac{1}{N_{s}}+u_{msr}^{\mathrm{MPE}}.$$

We specify the relationship between the MPE and the inverse of the sample size, reflecting that we expect reduced bias as the number of observations increases. In particular, the value of $\alpha_{m}^{\mathrm{MPE}}$ represents the value of the MPE to which the model approaches asymptotically with increasing sample size: testing whether or not this is statistically significant from 0 gives an indication of whether the estimator is consistent. Here, $u_{msr}^{\mathrm{MPE}}$ represents the error term from the regression. For the metrics that cannot be negative, we use the log‐function of the value as the dependent variable. With the log‐specification, differences in estimates are to be interpreted as percentage differences, as opposed to absolute differences.

## 6 Results and discussion

To begin, we consider the results from the smallest samples that we draw from the estimation set (5000 observations). Results from larger samples are analysed by way of the response surfaces which we present later. Table 3 is a key for the labels that we use for each model in discussion of the results.

**Table 3:** Key for model labels

OLS	Linear regression
LOGOLSHET	Transformed linear regression (logarithmically), heteroscedastic smearing factor
SQRTOLSHET	Transformed linear regression (square‐root), heteroscedastic smearing factor
GLMLOGP	GLM, log‐link, Poisson‐type family
GLMLOGG	GLM, log‐link, gamma‐type family
GLMSQRTP	GLM, square‐root link, Poisson‐type family
GLMSQRTG	GLM, square‐root link, gamma‐type family
LOGNORM	Log‐normal
GG	Generalized gamma
GB2LOG	Generalized beta of the second kind, log‐link
GB2SQRT	Generalized beta of the second kind, square‐root link
FMMLOGG	Two‐component finite mixture of gamma densities, log‐link
FMMSQRTG	Two‐component finite mixture of gamma densities, square‐root link
EEE	EEE method
CDEM	Conditional density approximation estimator (multinomial logit)
CDEO	Conditional density approximation estimator (ordered logit)

### 6.1 Estimation sample results

We first conduct tests of misspecification across the models used. Researchers use these tests to inform the specification of regressors, and the appropriateness of distributional assumptions, in particular the link function. Since we use the same regressors in all models, our tests are used to inform choices of distributional assumptions. The Pearson correlation coefficient test can detect whether there is a linear association between the estimated residuals and estimated conditional means, where the null hypothesis is no association. A lack of this kind of association suggests evidence against misspecification. It is also possible, however, that the relationship between the error and covariates is non‐linear, which this test cannot detect. Linear regression estimated by using OLS, by construction, generates residuals that are orthogonal to predicted costs, and so the Pearson test cannot be applied to this model. The Pearson test represents a simple test that is practically easy to implement and can be used to compare across different types of model. Researchers may wish to consider other means to choose between models; for instance Jones *et al*. (2014) compared Akaike information criterion and Bayesian information criterion scores, AIC and BIC. These provide a useful summary of the goodness of fit of the whole distribution on the scale of estimation, rather than the specification of the conditional mean function on the scale of interest in the Pearson test. In this context, when comparing parametric and semiparametric models, it is unclear how AIC and BIC could be calculated without imposing distributional assumptions on the methods that are not fully parametric.

Table 4 shows that, according to this test, there is less evidence of misspecification when the model is estimated by using a square‐root link function compared with other possible link functions, when all other distributional assumptions are the same. This is also the case in the GLM family of models, where the link and distribution functions can be flexibly estimated by using an EEE model, with results indicating that there is less evidence of misspecification with GLMSQRTP and GLMSQRTG than the flexible case (on average across replications with sample size 5000, the estimated *λ*‐coefficient in the EEE model was 0.28 with standard deviation of 0.07, indicating a link function between logarithmic and square root). Although the EEE model should be better specified on the scale of estimation (following, effectively, the transformation of the dependent variable), the retransformation may lead to increased evidence of misspecification on the scale of interest (levels of costs). Introducing more flexibility in terms of the whole distribution, generally, appears to have mixed effects on results from this test. In the case of LOGNORM and GLMLOGG which are special cases of GG, there is the least evidence of misspecification from the most complicated distribution among the three. There is also evidence of less misspecification with FMMLOGG compared with GLMLOGG, which it nests. Conversely, GG and LOGNORM are special cases of GB2LOG, for which there is the most evidence of misspecification among these three models. Looking at the rejection rates above for FMMSQRTG and GLMSQRTG, there is more evidence of misspecification in the more flexible case. Finally, the results from CDEM and CDEO are promising, with little evidence of misspecification compared with other models tested. This may be because there is no retransformation process onto the scale of interest for these models.

**Table 4:** Percentage of tests rejected at the 5% significance level, when all converged, 94 converged replications, sample size 5000

*Model*	*Pearson test*
	*rejection rate (%)*
OLS	—
LOGOLSHET	99
SQRTOLSHET	0
GLMLOGP	11
GLMLOGG	99
GLMSQRTP	0
GLMSQRTG	13
LOGNORM	95
GG	89
GB2LOG	96
GB2SQRT	85
FMMLOGG	85
FMMSQRTG	82
EEE	48
CDEM	7
CDEO	1

### 6.2 Validation set results

All tests in the previous section were carried out on the estimation sample. Given the practical implementation of the models that are considered here, a researcher may be more interested in how models perform in forecasting costs out of sample. Results based on the estimation sample may arise from overfitting the data. Therefore, our main focus is the forecasting performance out of sample, i.e. evaluation on the validation set.

We look first at performance of model predictions on the whole validation set. Then we consider how well the models forecast for different levels of covariates throughout the distribution, by analysing performance by decile of predicted costs. Finally, we analyse the out‐of‐sample performance with increasing sample size by constructing response surfaces.

Looking at the results in Table 5, where the four best performing models in each category (MPE, MAPE and RMSE) are in italics, it is clear that some of the most commonly used models—OLS, LOGOLSHET, GLMLOGG and LOGNORM—do not perform well on any metric. CDEM is among the models with top four performance in every category, illustrating the potential advantages of this approach for analysts concerned with any of bias, accuracy or goodness of fit. Generally, the results also indicate that a square‐root link function is the most appropriate of those featured. Interestingly, the Pearson test conducted on the estimation sample is shown here to perform well in discriminating between these competing models. This is encouraging given its ease of implementation and interpretation.

**Table 5:** Results of model performance, when all converged, sample size 5000, averaged across 94 replications

*Model*	*Bias MPE*	*Accuracy*	*Goodness‐of‐*
	*(*£*)*	*MAPE (*£*)*	*fit RMSE*
OLS	−1.56	1833.49	4475.49
LOGOLSHET	−140.53	1816.63	4960.08
SQRTOLSHET	*0.11*	1725.95	*4432.94*
GLMLOGP	−*1.44*	1748.43	4557.19
GLMLOGG	−147.33	1818.06	4984.86
GLMSQRTP	*0.26*	1704.77	*4426.24*
GLMSQRTG	46.71	*1689.28*	*4454.25*
LOGNORM	64.25	1734.10	4825.51
GG	44.60	1750.79	4754.22
GB2LOG	−63.96	1796.91	4873.13
GB2SQRT	134.84	*1686.48*	4483.35
FMMLOGG	−3.19	1758.06	4782.69
FMMSQRTG	121.80	*1690.28*	4477.10
EEE	−42.31	1727.28	4508.03
CDEM	*0.89*	*1683.40*	*4444.85*
CDEO	−10.13	1725.53	4474.84

In terms of bias, models which are mean preserving in sample also perform well out of sample in these results. This is evidenced by the strong performance of OLS, GLMLOGP and GLMSQRTP, with absolute levels of mean prediction error of £1.56, £1.44 and £0.26 respectively. All models with a square‐root link function underpredict costs on average, whereas some log‐link function models underpredict (LOGNORM and GG) and others overpredict on average (LOGOLSHET, GLMLOGP, GB2LOG and FMMLOGG). SQRTOLSHET and CDEM perform best and third best respectively, and worst performing is GLMLOGG, which overpredicts by £147.33 on average (5.64% of the population mean).

With respect to accuracy and goodness of fit, a clear message from the results is that the best performing link function is the square‐root function. The ordering of the other link functions varies. For accuracy the flexible link function of the EEE model is next best, followed by the log‐link function and then OLS. For goodness of fit OLS is second best, followed by EEE whereas the log‐link model is the worst. There is variation in performance among different models with the same link function, which we discuss next when considering the gains to increased flexibility. In addition, CDEM performs very well according to these criteria.

First we consider the gains in using a mixture of gamma distributions, over the nested single‐gamma‐distribution models. Looking at the results for GLMLOGG and FMMLOGG, the mixture improves forecasting performance in terms of bias, accuracy and goodness of fit. This is also observed in results from other sample sizes (see the on‐line appendix B). As discussed earlier, the gains to this increased flexibility are insufficient for results from FMMLOGG to perform better than relatively simple models using a square‐root link function (e.g. GLMSQRTP). Comparing results from GLMSQRTG with those from FMMSQRTG is more complicated, at sample size 5000; as seen in Table 5, we observe that GLMSQRTG performs better than FMMSQRTG in all metrics. FMMSQRTG performs better than GLMSQRTG, at larger samples, in terms of accuracy—with FMMSQRTG the best performing model of all 16 compared—but the nested single‐distribution case (GB2SQRTG) performs better, at all sample sizes, in terms of bias and goodness of fit (see appendix B).

Greater flexibility among the fully parametric models has an ambiguous effect on performance of forecasting means. GG is a limiting case of GB2 and its performance is better across all metrics. Conversely, LOGNORM, which is a special case of models GG and GB2, performs best of the three in terms of accuracy, the worst in terms of bias and second in terms of goodness of fit. Using GG or GB2LOG improves performance over special case GLMLOGG based on the MPE, MAPE and RMSE. Once again, the best of these four models performs worse than certain models with a square‐root link function. Comparing GLMSQRTG and GB2SQRT, we can see that not much is gained from introducing more parameters, since performance is worse for GB2SQRT than for GLMSQRTG except in the cases of accuracy at sample sizes 5000 and 10000 (the difference is small at all sample sizes analysed).

Crucially, these results consider only performance based on the mean, whereas some of these models can provide information on higher moments and on other features of the conditional distribution such as tail probabilities. This is a significant qualitative advantage of parametric models over models such as linear regression, where the models have been used to predict probabilities of lying beyond a threshold value, e.g. tail probabilities; see Jones *et al*. (2014) who found that the GG and LOGNORM distributions perform best for the threshold values they considered. We construct graphs of bias and accuracy by decile of predicted costs. This can be thought of as analysing the fit of models for the mean of distributions of costs conditionally on observed variables, since each decile of predicted costs represents a group of observations with certain values of covariates. In previous analysis, we have considered all observations as equal, but it is possible that a policy maker prioritizes the prediction error of certain observations over others. There is considerable interest in modelling the outcomes for high cost patients, since these can be responsible for large proportions of overall costs. The highest costs are likely to be found in the highest decile of predicted costs.

**Figure 3:**
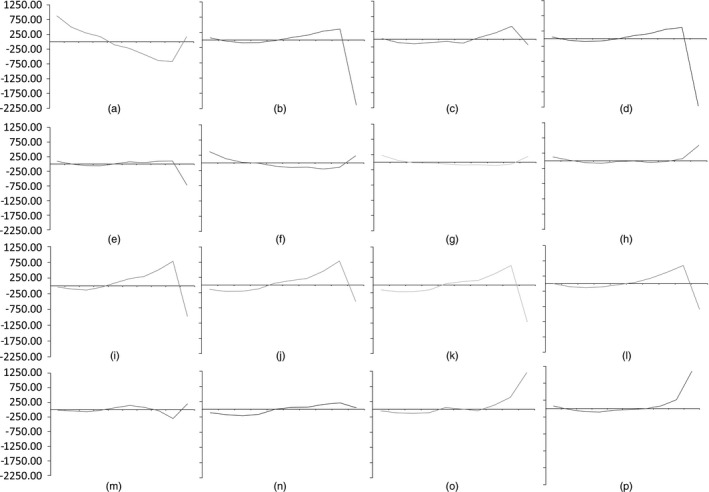
MPE by decile of fitted costs: (a) OLS; (b) LOGOLSHET; (c) GLMLOGP; (d) GLMLOGG; (e) EEE; (f) SQRTOLSHET; (g) GLMSQRTP; (h) GLMSQRTG; (i) LOGNORM; (j) GG; (k) GB2LOG; (l) RMMLOGG; (m) CDEM; (n) CDEO; (o) GB2SQRT; (p) RMMSQRTG

Fig. 3 shows that models with the same link function follow a largely similar pattern. Those, for example, with square‐root link functions underpredict in the decile of highest predicted costs, whereas log‐link models overpredict in the last decile. Results with other link functions—OLS, EEE, CDEM and CDEO—all have different patterns. Generally, the first decile ofpredicted costs from square‐root models are on average underpredictions (only GB2SQRToverpredicts in the smallest decile), which combined with the underpredicted last decile gives them a ‘u‐shaped’ line. The performance of each model varies across the deciles. SQRTOLSHET has a u‐shaped line and, although it performs best in predicting costs on average across all deciles, the performance for certain groups may be worse than that of other models. For example, CDEM performs slightly worse across all 10 deciles but has a smaller range of overpredictions and underpredictions. In terms of the highest decile of predicted costs, the model with the lowest MPE is CDEO, underestimating on average £48.96. Generally this decile tends to be the largest absolute MPE for models, with values as large as an average overprediction of £2211.47 in the case of GLMLOGG. Results for MAPE by decile of predicted cost are presented in the on‐line appendix B, where it is striking that the pattern across all models is very similar.

**Figure 4:**
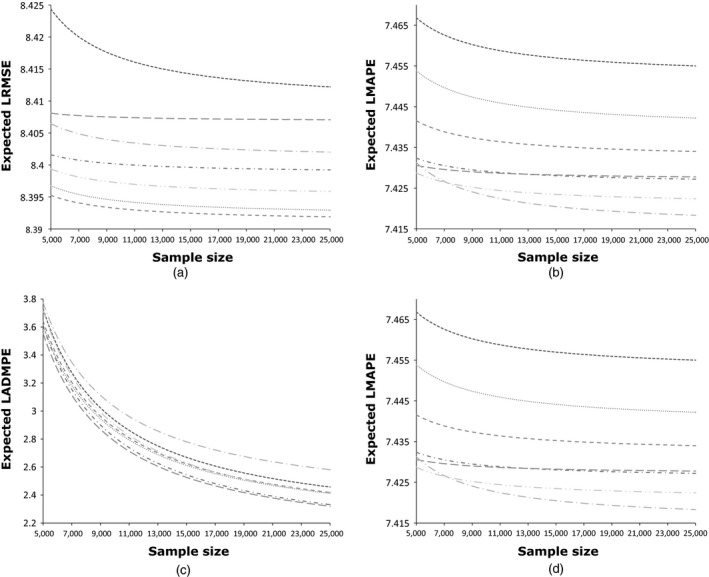
Response surfaces for (a) log(RMSE), (b) log(MAPE), (c) log(ADMPE) and (d) MPE against sample size, constructed by evaluating performance on ‘validation’ set: ……, SQRTOLSHET; **‐ ‐ ‐ ‐ ‐**, GLMLOGP; **– – –**, GLMSQRTP; **‐ · ‐ · ‐ · ‐**, GLMSQRTG; **– · –**, GB2SQRT; **— ·—**, FMMSQRTG; **· · — · ·**, CDEM

Fig. 4 displays the response surfaces constructed to analyse how each model's performance varied with increasing sample size for the subset of best performing models (those in italics in Table 5). We have already touched on this earlier when looking at results regarding accuracy between related distributions. The performance of most estimated models varies little as the sample sizes increase above 5000. There is some evidence that the variability of the MPE (measured by using the ADMPE) reduces as the sample size increases, although this happens at a similar rate across all models. Largely, though, the response surfaces for each model are parallel, indicating that the relative performance of models changes little. Further, the fact that they are flat represents evidence that performance does not change for each model with increasing sample size. The exception to this is that the performance of FMMSQRTG varies with increasing sample size: its accuracy improves, and its bias worsens. This suggests that this model behaves differently with samples as small as 5000 observations, possibly because of the number of parameters that are required. On the whole, though, from samples of 5000 observations or more, there is little evidence that more flexible models require more observations than less flexible models.

## 7 Conclusions

We have systematically evaluated the state of the art in regression models for healthcare costs, using administrative English hospital in‐patient data, employing a quasi‐Monte‐Carlo design to ensure rigour and drawing conclusions based on out‐of‐sample forecasting. We have compared recently adopted semiparametric and fully parametric regression methods that have never before been evaluated against one another, as well as comparing with regression methods that are now considered standard practice in modelling healthcare cost data.

Our results echo other studies, in that there is no single model that dominates in all respects: SQRTOLSHET is the best performing model in terms of bias and CDEM for accuracy, and in terms of goodness of fit the best performer is GLMSQRTP. This broadly corresponds to the results from the in‐sample Pearson test for model selection, which highlights its potential use as a simple means to discriminate between competing models. On the basis of the Pearson test results, researchers might have implemented other power transformations of the outcome in linear regression as well as in the link functions of the GLMs. Since the EEE model estimated that the appropriate link function was somewhere between a log‐ and square‐root function (on average 0.28 over all samples of 5000 observations), the researcher might have experimented with a cubic‐ or quartic‐root transformation. However, as square‐root models generally outperformed EEE models, there is no guarantee that results would have been better. Because there is no dominant model, the policy maker must weigh up these factors in arriving at their preferred model, based on their loss function over prediction errors. It is worth noting, however, that CDEM performs among the best four models for all three metrics. Another striking result is that four models that are commonly employed in regression methods for healthcare costs do not perform among the best four of any of the three metrics (OLS, LOGOLSHET, GLMLOGG and LOGNORM). Our analysis by decile shows the way in which models are sensitive to the choice of link function, with square‐root link functions underpredicting in the decile of highest predicted costs, and log‐link models overpredicting in the last decile. Finally, the response surfaces indicate that, on the whole, the more recent developments do not suffer because of the use of smaller sample sizes (from 5000 observations).
