# Supplementary material for: A quasi‐Monte‐Carlo comparison of parametric and semiparametric regression methods for heavy‐tailed and non‐normal data: an application to healthcare costs
Source: J R Stat Soc Ser A Stat Soc. 2015 Oct 15;179(4):951–74. doi: 10.1111/rssa.12141 (PMC5053270; doi:10.1111/rssa.12141)
Supplement: Supplementary file 1 [file RSSA-179-951-s001.pdf]

Appendix A

We use the variables shown in Table A1 to construct our regression models. They are based on the ICD10 chapters, which are given in Table A2.

Variable name	Variable description
epiA	Intestinal infectious diseases, Tuberculosis, Certain zoonotic bacterial diseases, Other bacterial diseases, Infections with a predominantly sexual mode of transmission, Other spirochaetal diseases, Other diseases caused by chlamydiae, Rickettsioses, Viral infections of the central nervous system, Arthropod-borne viral fevers and viral haemorrhagic fevers
epiB	Viral infections characterized by skin and mucous membrane lesions, Viral hepatitis, HIV disease, Other viral diseases, Mycoses, Protozoal diseases, Helminthiases, Pediculosis, acaiasis and other infestations, Sequelae of infectious and parasitic diseases, Bacterial, viral and other infectious agents, Other infectious diseases
epiC	Malignant neoplasms
epiD	In situ neoplasms, Benign neoplasms, Neoplasms of uncertain or unknown behaviour and III
epiE	IV
epiF	V
epiG	VI
epiH	VII and VIII
epiI	IX
epiJ	X
epiK	XI
epiL	XII
epiM	XIII
epiN	XIV
epiOP	XV and XVI
epiQ	XVII
epiR	XVIII
epiS	Injuries to the head, Injuries to the neck, Injuries to the thorax, Injuries to the abdomen, lower back, lumbar spine and pelvis, Injuries to the shoulder and upper arm, Injuries to the elbow and forearm, Injuries to the wrist and hand, Injuries to the hip and thigh, Injuries to the knee and lower leg, Injuries to the ankle and foot
epiT	Injuries involving multiple body regions, Injuries to unspecified part of trunk, limb or body region, Effects of foreign body entering through natural orifice, Burns and Corrosions, Frostbite, Poisoning by drugs, medicaments and biological substances, Toxic effects of substances chiefly nonmedicinal as to source, Other and unspecified effects of external causes, Certain early complications of trauma, Comlications of surgical and medical care, not elsewhere classified, Sequelae of injuries, of poisoning and of other consequences of external causes
epiU	XXII
epiV	Transport accidents
epiW	Falls, Exposure to inanimate mechanical forces, Exposure to animate mechanical forces, Accidental drowning and submersion, Other accidental threats to breathing, Exposure to electric current, radiation and extreme ambient air temperature and pressure
epiX	Exposure to smoke, fire and flames, Contact with heat and hot substances, Contact with venomous animals and plants, Exposure to forces of nature, Accidental poisoning by and exposure to noxious substances, Overexertion, travel and privation, Accidental exposure to other and unspecified factors, Intentional self-harm, Assault by drugs, medicaments and biological substances, Assault by corrosive substance, Assault by pesticides, Assault by gases and vapours, Assault by other specified chemicals and noxious substances, Assault by unspecified chemical or noxious substance, Assault by hanging, strangulation and suffocation, Assault by drowning and submersion, Assault by handgun discharge, Assault by rifle, shotgun and larger firearm discharge, Assault by other and unspecified firearm discharge, Assault by explosive material, Assault by smoke, fire and flames, Assault by steam, hot vapours and hot objects, Assault by sharp object
epiY	Assault by blunt object, Assault by pushing from high place, Assault by pushing or placing victim before moving object, Assault by crashing of motor vehicle, Assault by bodily force, Sexual assault by bodily force, Neglect and abandonment, Other maltreatment syndromes, Assault by other specified means, Assault by unspecified means, Event of undetermined intent, Legal intervention and operations of war, Complications of medical and surgical care, Sequelae of external causes of morbidity and mortality, Supplementary factors related to causes of morbidity and mortality classified else
epiZ	XXI

Table A1: Classification of morbidity characteristics

ICD10 codes beginning with U were dropped because there were no observations in the 6,164,114 used. Only a small number (3,170) were found of those beginning with P and so these were combined with those beginning with O - owing to the clinical similarities.

Chapter	Blocks	Title
I	A00-B99	Certain infectious and parasitic diseases
II	C00-D48	Neoplasms
III	D50-D89	Diseases of the blood and blood-forming organs and certain disorders involving the immune mechanism
IV	E00-E90	Endocrine, nutritional and metabolic diseases
V	F00-F99	Mental and behavioural disorders
VI	G00-G99	Diseases of the nervous system
VII	H00-H59	Diseases of the eye and adnexa
VIII	H60-H95	Diseases of the ear and mastoid process
IX	I00-I99	Diseases of the circulatory system
X	J00-J99	Diseases of the respiratory system
XI	K00-K93	Diseases of the digestive system
XII	L00-L99	Diseases of the skin and subcutaneous tissue
XIII	M00-M99	Diseases of the musculoskeletal system and connective tissue
XIV	N00-N99	Diseases of the genitourinary system
XV	O00-O99	Pregnancy, childbirth and the puerperium
XVI	P00-P96	Certain conditions originating in the perinatal period
XVII	Q00-Q99	Congenital malformations, deformations and chromosomal abnormalities
XVIII	R00-R99	Symptoms, signs and abnormal clinical and laboratory findings, not elsewhere classified
XIX	S00-T98	Injury, poisoning and certain other consequences of external causes
XX	V01-Y98	External causes of morbidity and mortality
XXI	Z00-Z99	Factors influencing health status and contact with health services
XXII	U00-U99	Codes for special purposes

Table A2: ICD10 chapter codes
