# Supplementary material for: A quasi‐Monte‐Carlo comparison of parametric and semiparametric regression methods for heavy‐tailed and non‐normal data: an application to healthcare costs
Source: J R Stat Soc Ser A Stat Soc. 2015 Oct 15;179(4):951–74. doi: 10.1111/rssa.12141 (PMC5053270; doi:10.1111/rssa.12141)
Supplement: Supplementary file 2 [file RSSA-179-951-s002.pdf]

1 Appendix B

Online only, not for print publication.

	Sample size		
**Model**	**10,000**	**50,000**	**100,000**
**OLS**	-	-	-
**LOGOLSHET**	100%	100%	100%
**SQRTOLSHET**	0%	44%	100%
**GLMLOGP**	46%	100%	100%
**GLMLOGG**	100%	100%	100%
**GLMSQRTP**	0%	13%	79%
**GLMSQRTG**	42%	100%	100%
**LOGNORM**	99%	100	100%
**GG**	99%	100%	100%
**GB2LOG**	100%	100%	100%
**GB2SQRT**	100%	100%	100%
**FMMLOGG**	98%	100%	100%
**FMMSQRTG**	97%	100%	100%
**EEE**	69%	100%	100%
**CDEM**	48%	100%	100%
**CDEO**	6%	89%	99%

Table B1: Results Pearson correlation coefficient tests (percentage rejected at 5% significance level)

	MPE (£)	MAPE (£)	RMSE
**OLS**	-6.19	1815.31	4460.42
**LOGOLSHET**	-149.73	1811.87	4938.27
**SQRTOLSHET**	-5.63	1715.94	4421.70
**GLMLOGP**	-6.77	1738.14	4522.67
**GLMLOGG**	-155.09	1813.78	4960.21
**GLMSQRTP**	-5.51	1699.16	4416.51
**GLMSQRTG**	41.14	1685.78	4447.57
**LOGNORM**	59.19	1732.46	4817.63
**GG**	43.26	1747.08	4743.14
**GB2LOG**	-68.96	1794.72	4865.08
**GB2SQRT**	131.03	1684.73	4480.02
**FMMLOGG**	-1.94	1747.21	4728.73
**FMMSQRTG**	136.06	1672.63	4461.85
**EEE**	-39.21	1716.71	4483.11
**CDEM**	-4.80	1677.85	4433.55
**CDEO**	-15.66	1724.60	4471.24

Table B2: Results of model performance, when all converged, sample size 10,000

	MPE (£)	MAPE (£)	RMSE
**OLS**	-1.81	1796.18	4449.72
**LOGOLSHET**	-147.59	1802.19	4906.22
**SQRTOLSHET**	-1.67	1703.75	4413.87
**GLMLOGP**	-1.27	1725.75	4495.21
**GLMLOGG**	-151.88	1804.52	4924.32
**GLMSQRTP**	-1.64	1690.68	4409.46
**GLMSQRTG**	44.49	1679.73	4442.24
**LOGNORM**	64.37	1726.74	4795.49
**GG**	52.19	1739.61	4721.18
**GB2LOG**	-59.70	1786.85	4838.97
**GB2SQRT**	135.16	1681.21	4478.19
**FMMLOGG**	-1.78	1739.02	4707.86
**FMMSQRTG**	139.06	1663.82	4453.58
**EEE**	-27.50	1703.97	4461.69
**CDEM**	-1.77	1671.65	4427.06
**CDEO**	-12.33	1721.03	4468.20

Table B3: Results of model performance, when all converged, sample size 50,000

	MPE (£)	MAPE (£)	RMSE
**OLS**	-1.38	1793.94	4448.34
**LOGOLSHET**	-145.55	1799.78	4894.66
**SQRTOLSHET**	-1.13	1702.17	4412.80
**GLMLOGP**	-0.23	1723.93	4491.04
**GLMLOGG**	-149.34	1801.91	4911.21
**GLMSQRTP**	-1.08	1689.60	4408.53
**GLMSQRTG**	45.22	1679.03	4441.85
**LOGNORM**	64.92	1725.98	4792.42
**GG**	52.96	1738.69	4718.07
**GB2LOG**	-59.08	1785.98	4835.64
**GB2SQRT**	135.08	1680.93	4477.82
**FMMLOGG**	0.10	1737.17	4702.38
**FMMSQRTG**	140.53	1662.90	4453.24
**EEE**	-23.27	1700.90	4456.40
**CDEM**	-1.26	1670.77	4426.08
**CDEO**	-11.73	1720.51	4467.82

Table B4: Results of model performance, when all converged, sample size 100,000

	**Decile of predicted cost**									
Model	1	2	3	4	5	6	7	8	9	10
**OLS**	878.41	498.24	301.60	177.47	-103.47	-218.87	-417.56	-627.31	-674.45	169.25
**LOGOLSHET**	72.19	-41.42	-90.06	-82.74	-18.47	81.12	158.80	294.32	356.78	-2135.90
**SQRTOLSHET**	366.18	137.92	18.07	-9.74	-104.61	-152.77	-140.83	-199.66	-147.44	233.38
**GLMLOGP**	23.56	-107.43	-149.27	-105.00	-71.83	-124.65	60.05	209.83	427.23	-176.98
**GLMLOGG**	50.81	-52.36	-96.95	-79.00	-12.05	94.98	162.95	304.74	364.94	-2211.47
**GLMSQRTP**	223.58	58.94	-24.00	-35.27	-59.78	-92.82	-82.67	-114.25	-60.64	189.14
**GLMSQRTG**	124.08	20.66	-59.16	-84.72	-22.72	-9.06	-54.07	-27.41	71.68	507.66
**LOGNORM**	-38.43	-99.80	-131.68	-43.98	97.28	230.40	300.73	510.15	782.57	-964.60
**GG**	-143.01	-210.11	-203.73	-128.22	59.81	146.80	217.61	454.21	789.59	-536.75
**GB2LOG**	-157.20	-222.93	-219.12	-154.65	45.15	111.74	155.70	371.60	642.29	-1212.01
**GB2SQRT**	-50.69	-114.56	-132.83	-109.30	59.73	6.65	-44.19	134.96	396.12	1202.57
**FMMLOGG**	-1.93	-98.99	-135.11	-105.46	-34.17	36.82	179.35	373.03	596.01	-841.44
**FMMSQRTG**	75.97	-25.76	-89.96	-103.93	-45.77	-31.70	-1.11	78.45	260.96	1100.74
**EEE**	91.79	0.36	-59.09	-62.56	6.34	75.45	46.98	94.32	103.54	-720.37
**CDEM**	-16.33	-40.35	-73.86	-18.31	69.14	144.93	79.88	-34.04	-302.29	200.10
**CDEO**	-111.98	-176.27	-213.31	-162.65	3.14	63.10	71.40	163.83	212.48	48.96

Table B5: Models' average mean prediction error (£) by decile of predicted cost at sample size 5,000

	Decile of predicted cost									
Model	1	2	3	4	5	6	7	8	9	10
**OLS**	821.88	469.46	293.66	173.65	-134.52	-228.16	-434.91	-634.77	-668.78	279.44
**LOGOLSHET**	59.60	-52.01	-106.49	-95.15	-30.01	67.37	156.75	280.35	346.95	-2124.82
**SQRTOLSHET**	333.52	114.76	-5.18	-16.99	-118.91	-169.77	-144.20	-200.53	-145.95	296.31
**GLMLOGP**	-7.46	-138.57	-177.80	-109.04	-69.16	-162.94	55.85	200.82	417.45	-76.94
**GLMLOGG**	40.74	-61.02	-109.44	-89.01	-26.68	83.26	160.16	288.70	354.38	-2192.10
**GLMSQRTP**	213.91	46.63	-39.07	-36.10	-76.04	-109.29	-90.19	-119.35	-71.38	225.44
**GLMSQRTG**	116.40	12.89	-73.70	-100.94	-47.57	12.06	-87.90	-33.00	71.34	541.61
**LOGNORM**	-44.86	-103.90	-143.50	-43.17	85.94	235.45	284.50	508.01	782.45	-968.86
**GG**	-144.37	-226.13	-201.47	-144.25	68.16	134.43	215.00	461.44	792.23	-522.18
**GB2LOG**	-159.65	-237.28	-216.89	-171.23	52.58	96.44	153.83	375.92	640.54	-1223.68
**GB2SQRT**	-55.62	-123.94	-128.14	-126.20	71.97	-5.73	-57.67	130.66	400.15	1204.90
**FMMLOGG**	-15.44	-115.07	-151.67	-104.54	-49.12	10.19	187.04	366.43	605.81	-753.02
**FMMSQRTG**	66.90	-35.99	-105.13	-132.67	-68.87	-24.54	-10.48	84.48	326.12	1260.66
**EEE**	85.26	-9.14	-74.40	-82.26	-25.88	88.86	17.44	97.27	116.52	-605.90
**CDEM**	-34.26	-43.05	-87.72	-22.54	58.40	143.35	68.13	-49.86	-328.54	248.16
**CDEO**	-119.90	-182.20	-225.87	-162.25	-17.17	60.67	64.90	165.18	215.17	44.94

Table B6: Models' average mean prediction error (£) by decile of predicted cost at sample size 10,000

	**Decile of predicted cost**									
Model	1	2	3	4	5	6	7	8	9	10
**OLS**	768.06	444.90	261.71	211.65	-162.65	-224.78	-436.43	-629.07	-636.96	384.46
**LOGOLSHET**	40.66	-63.18	-120.65	-98.74	-27.11	71.95	154.10	278.92	354.17	-2066.07
**SQRTOLSHET**	296.11	104.74	-34.85	5.55	-141.65	-177.56	-129.95	-175.76	-131.53	367.73
**GLMLOGP**	-37.97	-170.27	-203.77	-117.15	-50.17	-178.15	68.46	183.92	429.62	62.80
**GLMLOGG**	26.92	-70.31	-119.89	-90.86	-25.44	83.54	158.30	285.66	358.34	-2125.11
**GLMSQRTP**	198.14	37.23	-51.29	-24.78	-93.41	-109.47	-92.28	-101.31	-66.36	286.77
**GLMSQRTG**	104.24	7.93	-83.75	-100.93	-64.08	42.70	-117.01	-23.52	82.48	596.56
**LOGNORM**	-54.04	-111.30	-144.41	-41.15	81.71	236.05	269.16	515.50	789.18	-896.86
**GG**	-141.90	-238.99	-202.57	-154.60	77.50	113.44	230.59	469.88	804.80	-435.95
**GB2LOG**	-160.65	-245.80	-215.95	-181.97	61.39	72.92	174.47	382.37	657.11	-1140.59
**GB2SQRT**	-60.75	-130.80	-119.73	-129.98	82.93	-11.39	-68.85	138.36	413.63	1238.06
**FMMLOGG**	-34.22	-125.95	-161.88	-93.53	-49.81	-1.79	213.11	362.77	611.62	-738.07
**FMMSQRTG**	56.60	-38.19	-119.91	-132.29	-76.54	-11.73	-25.19	87.49	338.81	1311.47
**EEE**	76.23	-17.18	-84.60	-87.23	-43.18	103.90	-12.48	106.24	136.25	-453.13
**CDEM**	-47.07	-43.60	-101.08	-24.31	48.17	145.44	57.68	-56.54	-339.01	342.65
**CDEO**	-130.29	-190.45	-234.35	-156.66	-29.08	45.46	73.90	169.07	233.67	95.50

Table B7: Models' average mean prediction error (£) by decile of predicted cost at sample size 50,000

	Decile of predicted cost									
Model	1	2	3	4	5	6	7	8	9	10
**OLS**	761.33	441.25	263.68	211.50	-166.68	-225.62	-434.71	-628.76	-633.12	396.37
**LOGOLSHET**	38.30	-62.53	-125.03	-100.31	-27.79	72.07	150.17	280.03	355.76	-2036.25
**SQRTOLSHET**	290.94	106.72	-45.14	6.63	-146.40	-177.43	-131.87	-166.39	-127.88	378.95
**GLMLOGP**	-41.05	-173.23	-207.74	-119.94	-47.60	-178.91	70.02	178.29	432.38	85.42
**GLMLOGG**	25.07	-69.95	-123.63	-93.28	-24.34	81.28	155.48	285.85	360.55	-2090.44
**GLMSQRTP**	196.89	35.30	-54.10	-23.58	-98.07	-116.64	-90.84	-95.65	-64.14	299.60
**GLMSQRTG**	102.64	7.06	-85.09	-104.71	-66.86	47.18	-125.79	-18.43	85.42	610.59
**LOGNORM**	-54.69	-112.28	-143.09	-42.46	81.70	237.09	264.89	516.00	789.82	-887.70
**GG**	-140.56	-241.55	-200.69	-157.87	76.96	109.26	233.94	468.15	806.01	-423.72
**GB2LOG**	-159.90	-247.54	-214.15	-185.09	59.92	65.76	180.76	380.80	659.11	-1130.20
**GB2SQRT**	-61.84	-131.53	-119.32	-130.27	81.62	-11.40	-72.45	139.53	414.41	1241.97
**FMMLOGG**	-35.47	-126.45	-164.61	-88.71	-57.36	-9.50	218.23	360.09	615.49	-710.66
**FMMSQRTG**	54.01	-39.31	-124.91	-136.39	-76.82	-10.32	-31.55	92.74	344.52	1333.18
**EEE**	76.77	-19.28	-85.65	-91.64	-45.13	95.20	-15.34	106.00	141.78	-395.60
**CDEM**	-48.70	-44.60	-101.13	-24.65	46.88	145.68	55.88	-58.64	-339.60	356.41
**CDEO**	-131.91	-189.53	-236.78	-153.91	-33.74	44.92	73.75	171.04	236.17	102.71

Table B8: Models' average mean prediction error (£) by decile of predicted cost at sample size 100,000

	Decile of predicted cost									
Model	1	2	3	4	5	6	7	8	9	10
**OLS**	879.77	575.57	639.97	864.80	1120.42	1530.58	1953.91	2437.88	3165.22	5167.22
**LOGOLSHET**	432.16	567.71	690.35	825.97	1038.71	1341.88	1702.04	2181.12	2896.97	6490.37
**SQRTOLSHET**	472.78	530.79	673.13	878.29	1104.90	1427.97	1839.97	2275.21	2969.18	5088.17
**GLMLOGP**	472.76	624.62	749.98	916.79	1127.15	1354.09	1737.56	2192.54	2871.79	5438.02
**GLMLOGG**	431.11	564.87	686.22	824.57	1032.54	1341.37	1692.92	2175.93	2895.86	6536.24
**GLMSQRTP**	410.47	531.24	669.20	853.52	1093.50	1405.85	1801.24	2242.47	2942.28	5098.81
**GLMSQRTG**	407.94	522.60	655.95	849.47	1084.61	1415.75	1778.61	2244.43	2950.61	4983.76
**LOGNORM**	500.51	580.15	657.62	833.68	1056.19	1325.01	1625.88	2065.70	2789.91	5907.09
**GG**	601.19	640.44	733.21	888.59	1143.62	1368.59	1622.82	2046.71	2761.63	5701.97
**GB2LOG**	607.97	648.02	740.91	899.20	1163.18	1389.65	1633.55	2067.98	2790.67	6028.71
**GB2SQRT**	544.30	584.86	701.04	922.90	1220.87	1420.87	1672.45	2142.12	2837.97	4818.24
**FMMLOGG**	455.63	598.45	722.83	869.04	1070.73	1338.32	1691.78	2150.84	2837.05	5846.93
**FMMSQRTG**	422.12	556.24	696.00	879.97	1116.32	1413.42	1772.12	2217.32	2910.98	4919.30
**EEE**	415.25	531.62	651.85	836.66	1049.22	1386.31	1739.97	2225.07	2966.02	5471.84
**CDEM**	476.96	555.78	639.96	780.26	1019.69	1335.12	1683.51	2213.41	3013.70	5116.58
**CDEO**	576.63	626.78	717.86	889.49	1142.60	1409.13	1694.02	2146.64	2915.21	5137.76

Table B9: Models' average mean absolute prediction error (£) by decile of predicted cost at sample size 5,000

	Decile of predicted cost									
Model	1	2	3	4	5	6	7	8	9	10
**OLS**	823.15	555.90	631.90	860.48	1101.13	1540.49	1950.52	2427.09	3139.43	5123.35
**LOGOLSHET**	431.30	567.50	688.57	816.60	1031.93	1331.88	1709.15	2170.82	2891.03	6480.89
**SQRTOLSHET**	451.16	528.18	664.82	878.72	1089.47	1422.35	1840.52	2266.38	2951.12	5067.67
**GLMLOGP**	477.64	625.26	746.17	906.93	1131.35	1337.55	1734.70	2182.37	2859.00	5381.39
**GLMLOGG**	431.00	565.77	684.20	819.89	1023.22	1333.50	1701.53	2167.19	2890.53	6521.88
**GLMSQRTP**	405.98	531.14	661.24	856.03	1079.96	1400.72	1803.69	2236.63	2930.65	5086.55
**GLMSQRTG**	406.69	523.08	654.88	846.33	1064.11	1435.75	1765.19	2241.50	2942.36	4978.86
**LOGNORM**	502.29	582.93	648.20	841.26	1049.65	1324.72	1618.63	2064.72	2789.15	5903.89
**GG**	607.39	633.99	727.07	887.22	1142.93	1353.45	1624.78	2046.48	2756.94	5691.36
**GB2LOG**	614.49	642.18	734.34	900.43	1159.46	1376.05	1637.34	2068.12	2787.27	6028.33
**GB2SQRT**	549.51	580.31	696.52	918.68	1229.69	1411.57	1668.49	2140.93	2835.99	4816.42
**FMMLOGG**	460.53	598.19	720.21	864.41	1059.81	1314.83	1694.76	2139.13	2823.88	5797.27
**FMMSQRTG**	424.65	554.36	697.88	868.73	1084.75	1416.72	1760.39	2185.57	2866.85	4867.40
**EEE**	413.78	534.75	653.24	833.36	1025.46	1399.82	1727.13	2217.48	2948.43	5414.48
**CDEM**	478.12	554.96	638.92	778.62	1017.52	1334.62	1678.98	2206.38	3002.68	5088.50
**CDEO**	581.02	628.72	709.23	898.91	1127.09	1411.07	1695.33	2146.62	2915.49	5133.30

Table B10: Models' average mean absolute prediction error (£) by decile of predicted cost at sample size 10,000

	Decile of predicted cost									
Model	1	2	3	4	5	6	7	8	9	10
**OLS**	768.85	541.26	610.06	881.75	1069.10	1551.55	1933.73	2409.88	3113.74	5082.23
**LOGOLSHET**	429.58	561.83	688.53	807.53	1024.78	1329.72	1704.85	2164.61	2880.53	6430.87
**SQRTOLSHET**	422.32	539.92	640.41	893.53	1058.08	1413.50	1840.22	2257.59	2931.13	5041.73
**GLMLOGP**	480.08	618.62	756.96	878.99	1138.11	1328.83	1746.09	2149.72	2853.08	5307.87
**GLMLOGG**	431.81	560.11	684.30	814.80	1015.15	1331.36	1698.38	2162.99	2879.88	6467.43
**GLMSQRTP**	396.93	534.22	650.66	866.29	1055.36	1395.82	1798.02	2233.11	2914.62	5062.77
**GLMSQRTG**	405.92	521.15	647.28	852.27	1039.32	1459.18	1744.01	2236.95	2932.55	4959.75
**LOGNORM**	500.44	584.59	645.16	844.44	1053.92	1318.84	1609.03	2065.50	2783.57	5862.68
**GG**	611.60	629.46	722.95	877.98	1144.29	1330.65	1638.31	2042.62	2750.67	5648.39
**GB2LOG**	619.63	635.77	732.23	894.72	1155.89	1352.95	1654.53	2062.14	2782.81	5978.79
**GB2SQRT**	553.94	575.79	690.26	919.19	1235.06	1399.53	1658.93	2139.57	2830.54	4810.14
**FMMLOGG**	461.64	596.10	713.92	864.25	1043.18	1293.90	1704.38	2120.48	2810.22	5783.08
**FMMSQRTG**	426.90	554.34	687.22	870.68	1059.59	1428.87	1739.51	2170.59	2851.74	4849.70
**EEE**	412.88	535.05	649.33	837.32	1004.28	1413.33	1708.59	2212.19	2930.73	5337.10
**CDEM**	474.76	556.18	640.39	776.69	1017.12	1337.97	1671.41	2201.47	2990.46	5050.98
**CDEO**	584.29	627.26	708.12	905.02	1116.05	1402.84	1704.46	2140.35	2913.49	5109.27

Table B11: Models' average mean absolute prediction error (£) by decile of predicted cost at sample size 50,000

	Decile of predicted cost									
Model	1	2	3	4	5	6	7	8	9	10
**OLS**	761.93	538.22	613.63	882.50	1061.12	1553.78	1932.49	2407.91	3111.01	5077.11
**LOGOLSHET**	430.25	562.48	687.36	805.11	1025.77	1329.85	1703.25	2165.65	2877.75	6411.24
**SQRTOLSHET**	418.79	544.34	632.39	898.00	1053.61	1411.66	1839.24	2258.54	2928.42	5037.66
**GLMLOGP**	481.31	616.64	760.43	872.17	1138.80	1329.53	1749.36	2141.91	2853.14	5296.89
**GLMLOGG**	432.92	560.49	683.34	812.21	1017.80	1329.84	1697.54	2163.70	2877.59	6444.67
**GLMSQRTP**	396.72	535.84	647.29	869.59	1051.08	1391.81	1798.97	2234.50	2912.49	5058.61
**GLMSQRTG**	407.62	518.79	648.16	851.77	1037.56	1462.26	1739.84	2237.83	2931.44	4956.07
**LOGNORM**	499.05	585.58	645.14	842.99	1054.22	1319.81	1607.29	2065.35	2782.88	5858.31
**GG**	613.13	629.43	722.41	875.78	1142.98	1327.14	1643.17	2040.20	2749.96	5643.64
**GB2LOG**	621.73	634.84	732.10	893.66	1152.43	1347.92	1661.59	2060.34	2782.50	5973.69
**GB2SQRT**	555.68	575.06	688.65	919.95	1234.31	1399.84	1656.56	2140.01	2829.95	4810.15
**FMMLOGG**	462.06	595.23	714.82	867.91	1040.55	1288.38	1707.44	2118.45	2808.24	5769.40
**FMMSQRTG**	426.74	555.77	686.13	872.24	1057.40	1432.20	1734.65	2168.91	2850.22	4845.77
**EEE**	413.77	533.85	651.76	836.46	1006.16	1408.20	1710.56	2210.26	2926.58	5312.42
**CDEM**	474.63	554.41	641.63	776.70	1016.61	1339.16	1670.01	2201.30	2988.91	5045.26
**CDEO**	584.83	627.99	705.53	907.38	1110.80	1404.08	1705.17	2140.17	2913.84	5106.01

Table B12: Models' average mean absolute prediction error (£) by decile of predicted cost at sample size 100,000

	**Response surface regressions**			
**Regression coefficient**	**MPE**	**LMAPE**	**LRMSE**	**LADMPE**
α	-2.46<br>(1.87)	7.49<br>(0.00)	8.40<br>(0.00)	2.11<br>(0.09)
β	-3524.10<br>(32871.71)	113.56<br>(15.16)	31.89<br>(1.98)	7432.02<br>(773.24)

Table B13: Regression coefficients for OLS response surface regressions

	**Response surface regressions**			
**Regression coefficient**	**MPE**	**LMAPE**	**LRMSE**	**LADMPE**
α	-148.13<br>(2.31)	7.50<br>(0.00)	8.50<br>(0.00)	2.29<br>(0.09)
β	27441.85<br>(40826.75)	47.30<br>(11.91)	64.37<br>(18.59)	8147.63<br>(717.13)

Table B14: Regression coefficients for LOGOLSHET response surface regressions

	**Response surface regressions**			
**Regression coefficient**	**MPE**	**LMAPE**	**LRMSE**	**LADMPE**
α	-2.44<br>(1.88)	7.44<br>(0.00)	8.39<br>(0.00)	2.11<br>(0.09)
β	4161.85<br>(33067.30)	72.66<br>(10.43)	23.83<br>(1.29)	7496.99<br>(828.20)

Table B15: Regression coefficients for SQRTOLSHET response surface regressions

	Response surface regressions			
**Regression coefficient**	**MPE**	**LMAPE**	**LRMSE**	**LADMPE**
α	-1.75<br>(1.95)	7.45<br>(0.00)	8.41<br>(0.00)	2.14<br>(0.09)
β	-8381.67<br>(35203.29)	73.77<br>(9.66)	76.03<br>(7.89)	7947.14<br>(721.92)

Table B16: Regression coefficients for GLMLOGP response surface regressions

	Response surface regressions			
**Regression coefficient**	**MPE**	**LMAPE**	**LRMSE**	**LADMPE**
α	-151.94<br>(2.37)	7.50<br>(0.00)	8.50<br>(0.00)	2.37<br>(0.08)
β	12368.49<br>(41957.46)	44.87<br>(12.24)	72.04<br>(19.88)	7680.98<br>(735.76)

Table B17: Regression coefficients for GLMLOGG response surface regressions

	Response surface regressions			
**Regression coefficient**	**MPE**	**LMAPE**	**LRMSE**	**LADMPE**
α	-2.40<br>(1.87)	7.43<br>(0.00)	8.39<br>(0.00)	2.11<br>(0.09)
β	4768.78<br>(33017.63)	47.11<br>(8.07)	21.02<br>(1.08)	7661.16<br>(707.81)

Table B18: Regression coefficients for GLMSQRTP response surface regressions

	Response surface regressions			
**Regression coefficient**	**MPE**	**LMAPE**	**LRMSE**	**LADMPE**
α	43.86<br>(1.74)	7.43<br>(0.00)	8.40<br>(0.00)	2.01<br>(0.09)
β	6300.86<br>(30958.31)	32.27<br>(6.43)	14.78<br>(1.35)	8004.18<br>(702.89)

Table B19: Regression coefficients for GLMSQRTG response surface regressions

	Response surface regressions			
**Regression coefficient**	**MPE**	**LMAPE**	**LRMSE**	**LADMPE**
α	63.69<br>(1.55)	7.45<br>(0.00)	8.47<br>(0.00)	1.92<br>(0.08)
β	-6348.01<br>(27826.75)	24.95<br>(6.92)	36.18<br>(9.83)	8009.81<br>(687.22)

Table B20: Regression coefficients for LOGNORM response surface regressions

	Response surface regressions			
**Regression coefficient**	**MPE**	**LMAPE**	**LRMSE**	**LADMPE**
α	52.17<br>(1.70)	7.46<br>(0.00)	8.46<br>(0.00)	1.89<br>(0.10)
β	-47610.97<br>(30514.46)	36.78<br>(7.41)	40.14<br>(8.65)	8123.00<br>(830.41)

Table B21: Regression coefficients for GG response surface regressions

	Response surface regressions			
**Regression coefficient**	**MPE**	**LMAPE**	**LRMSE**	**LADMPE**
α	-60.42<br>(2.21)	7.49<br>(0.00)	8.48<br>(0.00)	2.29<br>(0.09)
β	-30592.30<br>(41448.16)	32.25<br>(10.48)	40.50<br>(12.11)	7699.85<br>(733.74)

Table B22: Regression coefficients for GB2LOG response surface regressions

	Response surface regressions			
**Regression coefficient**	**MPE**	**LMAPE**	**LRMSE**	**LADMPE**
α	134.39<br>(1.62)	7.43<br>(0.00)	8.41<br>(0.00)	2.01<br>(0.08)
β	-4550.79<br>(29380.16)	17.68<br>(5.35)	6.36<br>(1.41)	7719.11<br>(688.62)

Table B23: Regression coefficients for GB2SQRT response surface regressions

	Response surface regressions			
**Regression coefficient**	**MPE**	**LMAPE**	**LRMSE**	**LADMPE**
α	-0.61<br>(2.70)	7.46<br>(0.00)	8.45<br>(0.00)	2.44<br>(0.08)
β	-13166.18<br>(50627.97)	60.05<br>(14.09)	83.16<br>(19.16)	8279.92<br>(757.08)

Table B24: Regression coefficients for FMMLOGG response surface regressions

	Response surface regressions			
**Regression coefficient**	**MPE**	**LMAPE**	**LRMSE**	**LADMPE**
α	142.12<br>(2.85)	7.42<br>(0.00)	8.40<br>(0.00)	2.28<br>(0.07)
β	-93880.38<br>(67740.82)	80.92<br>(20.79)	27.85<br>(6.12)	7461.19<br>(779.31)

Table B25: Regression coefficients for FMMSQRTG response surface regressions

	Response surface regressions			
**Regression coefficient**	**MPE**	**LMAPE**	**LRMSE**	**LADMPE**
α	-25.08<br>(2.35)	7.44<br>(0.00)	8.40<br>(0.00)	2.36<br>(0.08)
β	-96992.48<br>(44199.43)	78.57<br>(12.04)	58.99<br>(7.25)	7962.63<br>(726.73)

Table B26: Regression coefficients for EEE response surface regressions

	Response surface regressions			
**Regression coefficient**	**MPE**	**LMAPE**	**LRMSE**	**LADMPE**
α	-2.52<br>(1.86)	7.42<br>(0.00)	8.39<br>(0.00)	2.08<br>(0.08)
β	9424.60<br>(32387.59)	39.46<br>(6.22)	22.01<br>(1.16)	8165.22<br>(685.22)

Table B27: Regression coefficients for CDEM response surface regressions

	Response surface regressions			
**Regression coefficient**	**MPE**	**LMAPE**	**LRMSE**	**LADMPE**
α	-13.02<br>(1.91)	7.45<br>(0.00)	8.40<br>(0.00)	2.16<br>(0.09)
β	6579.60<br>(33749.36)	15.36<br>(6.44)	8.26<br>(1.00)	7808.60<br>(741.31)

Table B28: Regression coefficients for CDEO response surface regressions
